# Retraction Note: Impact of artificial intelligence on human loss in decision making, laziness and safety in education

**DOI:** 10.1057/s41599-026-06602-8

**Published:** 2026-02-03

**Authors:** Sayed Fayaz Ahmad, Heesup Han, Muhammad Mansoor Alam, Mohd. Khairul Rehmat, Muhammad Irshad, Marcelo Arraño-Muñoz, Antonio Ariza-Montes

**Affiliations:** 1 Institute of Business Management, Karachi, Pakistan; 2 Sejong University, Seoul, Korea; 3 Riphah International University, Islamabad, Pakistan; 4 Universiti Kuala Lumpur, Kuala Lumpur, Malaysia; 5 University of Gwadar, Gwadar, Pakistan; 6 Universidad de Playa Ancha, Valparaiso, Chile; 7 Universidad Loyola Andalucía, Córdoba, Spain

Retraction Note to: *Humanities and Social Sciences Communications* 10.1057/s41599-023-01787-8, published online 09 June 2023

The Editor retracts this article due to concerns regarding insufficient ethical approval for the research involving human participants. The study collected data in both Pakistan and China, yet no authors were affiliated with a Chinese institution, and the authors provided only an approval document from the University of Gwadar, Pakistan. Thus, it is not clear how and with what oversight the Chinese data were collected, and the Editors have therefore determined that the documents provided do not meet the journal’s requirements for human research ethics.

All the authors disagree with this retraction.

